# Effect of heterogeneity on failure of natural rock samples

**DOI:** 10.1038/s41598-020-71780-7

**Published:** 2020-09-07

**Authors:** Taqi Alzaki, Saud Al-Dughaimi, Arqam Muqtadir, Mohamed E. Kandil, Jack Dvorkin

**Affiliations:** College of Petroleum Engineering and Geosciences (CPG), King Fahd University of Petroleum and Minerals (KFUPM), Dhahran, Saudi Arabia

**Keywords:** Geophysics, Solid Earth sciences, Materials science

## Abstract

A carbonate sample extracted from the depth of about 10 kft was subjected to uniaxial loading while the confining stress remained constant. Post-experiment inspection of the sample showed an inclined crack at an angle less than 20° to the horizontal. This subhorizontal crack orientation was contrary to the expected 45° inclination, the plane of the maximum shear stress. Coincidentally, as shown by CT-scan prior to loading, there was a boundary between two layers of different density inside the sample located almost exactly where the crack appeared. This density difference has arguably translated into the contrast in the elastic properties at the boundary. The hypothesis is that because of this elastic heterogeneity, an incipient crack developed at the boundary due to the unavoidable tensile stressing of the sample as it was brought to the benchtop from its original state of high confining stress at depth. Controlled uniaxial compression made the sample slip along this crack, which then developed into a prominent feature. This assumption was corroborated by a numerical experiment showing a strong von Mises stress concentration at the elastic contrast boundary during hydrostatic tensile loading. Another sample, from the same formation, but without strong density heterogeneity, exhibited a classic 45° crack after uniaxial loading. These results provide a novel and important insight into the mechanics, breakage, and strength of natural rock.

## Introduction

The predominant mode of the failure of natural rock is fracturing. The amount of past work on this topic is massive. Classic papers and monographs^1–4^ have been followed by a plethora of recent publications describing experimental results, both physical and computational. Experiments on artificial layered rock samples^5^ indicate that failure cracks often appear along layer interfaces. Similar results are reported for slate samples^6^.

A “single plane of weakness” rock failure theory^7^ based on earlier observations^8^ refers to an isotropic and homogeneous material cut through by a plane with reduced shear strength. Failure under axial load occurs on this plane. Later papers, such as^9^, invoke this theory as well.

By and large, the abovementioned references, together with other numerous publications, experimental and theoretical, link rock failure to the presence of meso-scale anisotropy and inhomogeneity in rock samples.

**Figure 1:**
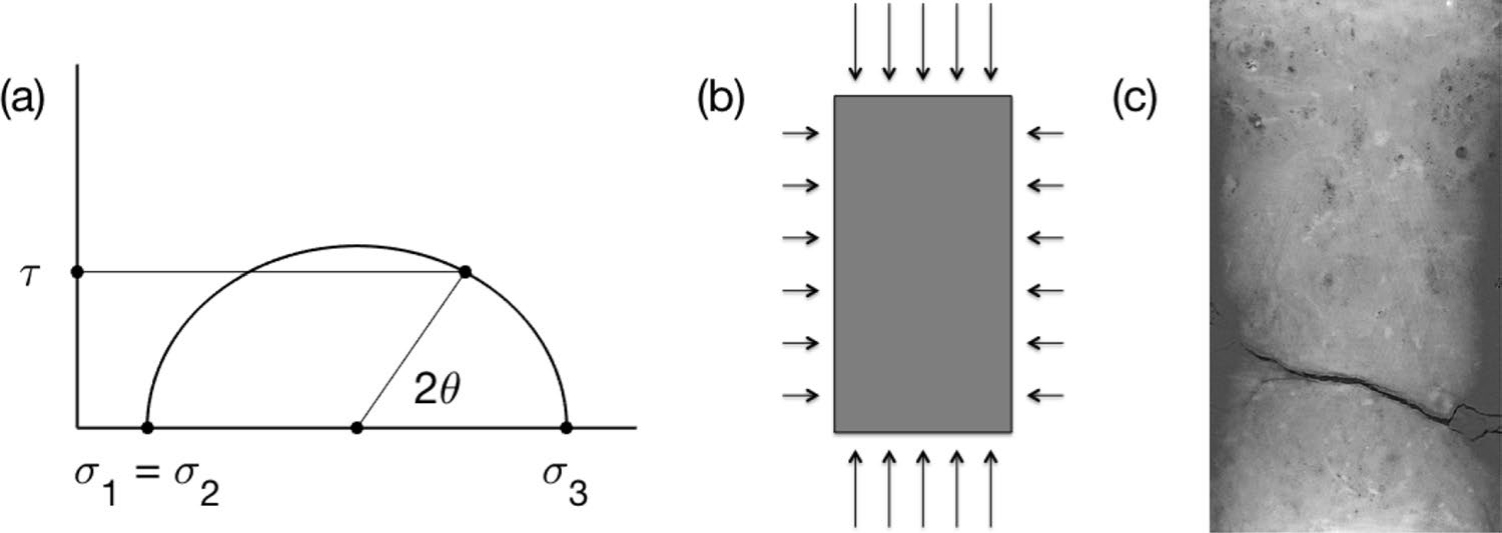
(**a**) Mohr circle for shear stress *τ* at an angle *θ* to the horizontal during vertical loading *σ*_3_ with equal lateral stresses *σ*_1_ = *σ*_2_. (**b**) A schema of uniaxial loading. The arrows show the stress boundary conditions: the lateral (confining) stress as denoted by the horizontal arrows is smaller than the axial stress as denoted by the vertical arrows. (**c**) Carbonate sample post uniaxial loading with the subhorizontal crack (black), a 2D vertical section taken along the central axis of the 3D CT-scan volume.

In contrast, for an isotropic and homogeneous rock, classic strength of materials theory gives the following expression for the shear stress $\tau$ along the plane inclined at angle $\theta$ to the horizontal under uniaxial loading where the principal vertical stress $\sigma_{3}$ exceeds the two equal horizontal stresses $\sigma_{1}$ = $\sigma_{2}$:

1 $$\tau = \frac{{\sigma_{3} - \sigma_{1} }}{2}\sin 2\theta ,$$

a formula conveniently visualized by the Mohr circle (Fig. 1).

According to Eq. (1), the maximum shear stress occurs on the plane cutting the sample at 45° to the horizontal. However, a carbonate sample under examination subjected to uniaxial loading developed a subhorizontal crack inclined at about 20° (Fig. 1c). What could have caused such a counterintuitive behavior?

### Tests and results

Carbonate samples C23 and C8 extracted from a well drilled in a Saudi oil field were cleaned and dried in the laboratory. Their dynamic Poisson’s ratio $\nu$ and Young’s modulus $E$ were computed from the bulk density $\rho_{b}$ and the P- and S-wave velocities, $V_{p}$ and $V_{s}$, measured at 30 MPa hydrostatic stress as

2 $$\nu = \frac{1}{2}\frac{{(V_{p} /V_{s} )^{2} - 2}}{{(V_{p} /V_{s} )^{2} - 1}};\quad E = 2\rho_{b} V_{s}^{2} (1 + \nu )$$

and are listed, together with the depth of their original location, in Table 1.

**Table 1:** Properties of carbonate samples under examination.

Sample	Depth (ft)	Porosity	Permeability (mD)	*ρ*_*b*_ (g/cc)	*E* (GPa)	*ν*
C23	10,179.0	0.1867	3.210	2.220	26.340	0.196
C8	10,118.2	0.1247	0.664	2.363	49.586	0.274

**Figure 2:**
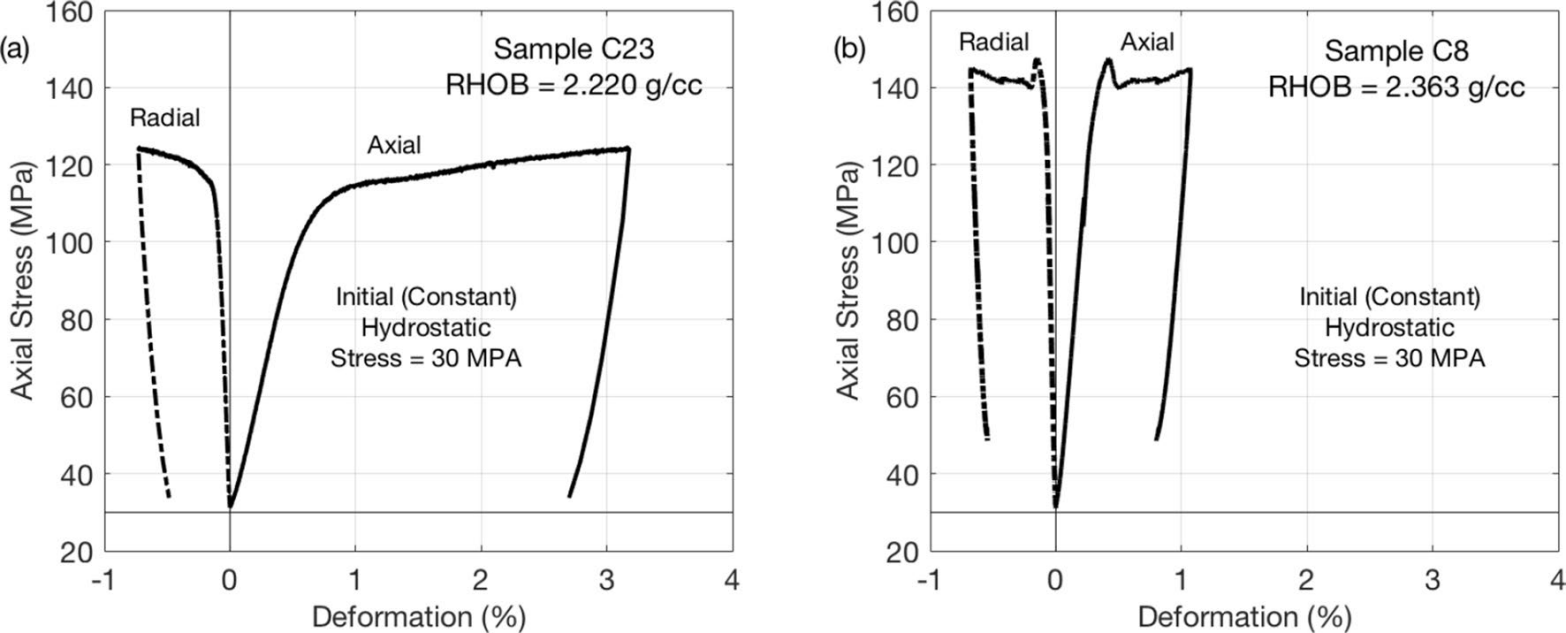
Stress versus axial deformation for Samples C23 (**a**) and C8 (**b**). The radial deformation (dash-dotted curves) is negative (thickening), while the axial deformation (solid curves) is positive (shortening).

Both samples were first hydrostatically loaded to 30 MPa. Afterwards, the vertical stress was gradually increased until failure, with the confining stress remaining constant. Finally, after a period of post-failure deformation, the samples were axially unloaded to the initial hydrostatic stress 30 MPa. All tests were performed using a load-frame Autolab-1500 supplied by New England Research. The elastic-wave velocities were measured on room-dry samples using ultrasonic transducers with 750 kHz central frequency. The axial and radial deformations were measured by linear variable differential transformers (LVDT). The results of these tests are shown in Fig. 2.

In both tests, observed was a textbook behavior with almost linearly elastic deformation followed by failure, plastic deformation, and elastic unloading. The unloading curves essentially parallel the loading curves. The post-yield flow time for C23 was 30 min, while it was 8 min for C8. This explains why the post-yield deformation in C23 was about three times that in C8.

### First sample: post-test and pre-test images

**Figure 3:**
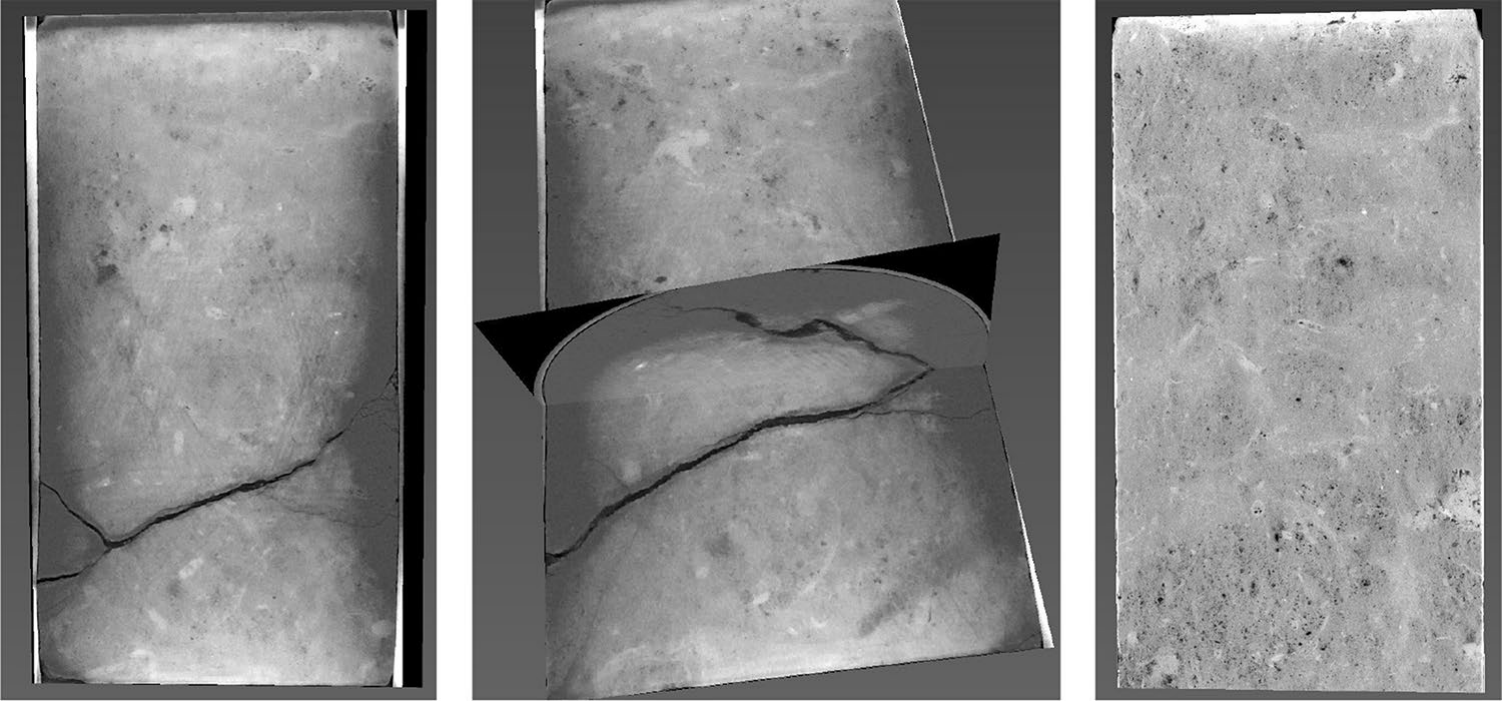
Vertical slices along the central axis of a 3D CT scan of C23 after the loading experiment shown at different image orientations (first two frames) and a pre-test vertical slice of a 3D CT scan of the same sample (third frame). The sample is 1 inch in diameter and 2 inches long.

Post-test CT-scan images (vertical slices of the original 3D images taken along the central axis) for sample C23 are shown in Fig. 3. The major crack in the lower half of the sample is inclined at about 20° to the horizontal and surrounded by minor cracks at the circumference of the sample.

The pre-test image (also a vertical slice of the original 3D image taken along the central axis) shown in the same figure, exhibits a clear CT-value contrast at the position where the crack later developed. These CT values indicate a density and, hence, porosity contrast and, as a result, the elastic moduli contrast with the stiffer part of the sample located above its softer part.

## Methods

The hypothesis is that an incipient subtle crack developed at the boundary as the sample was brought from its natural depth of about 10 kft to the ambient conditions at the benchtop. This crack became the “plane of weakness” along which the sample failed during the experiment.

Indeed, the original confining stress at depth was on the order of 30 to 40 MPa. Upon its extraction, the sample was inadvertently subjected to tensile hydrostatic stress as the stress was reduced from its original value to zero. As a result, an incipient crack appeared at the boundary inside the sample and then developed into a major fracture during the uniaxial loading.

To test this hypothesis, a digital cylindrical sample with a plane boundary cutting it at about 20° to the horizontal was created. The Young’s moduli $E$ above and below this discontinuity were assigned 56 and 36 GPa, respectively, while the Poisson’s ratio $\nu$ was the same 0.3.

The density distribution inside the model was assigned based on the CT-value contrast shown in Fig. 3. The total porosity was computed from density and using the mass-balance law by assuming that the mineral is pure calcite with density 2.71 g/cc. Finally, this porosity contrast was translated into the elastic-moduli contrast by assuming the stiff-rock effective-medium model described in, e.g.,^10^. This model accurately describes the velocity–porosity wireline data from the well where the samples were extracted from (see^11^).

Uniform hydrostatic tension of 30 MPa was applied to this digital object and the von Mises stress

3 $$\sigma_{VM} = \sqrt {\frac{1}{2}\left[ {(\sigma_{xx} - \sigma_{yy} )^{2} + (\sigma_{yy} - \sigma_{zz} )^{2} + (\sigma_{zz} - \sigma_{xx} )^{2} } \right] + 3\left( {\tau_{xy}^{2} + \tau_{yz}^{2} + \tau_{zx}^{2} } \right)}$$

was computed using the finite elements method (FEM). These computations were done using a commercial FEM package COMSOL, where the digital model was meshed by triangular elements fine enough to delineate the boundary of the elastic contrast and resolve the ensuing stress concentration at this boundary.

**Figure 4:**
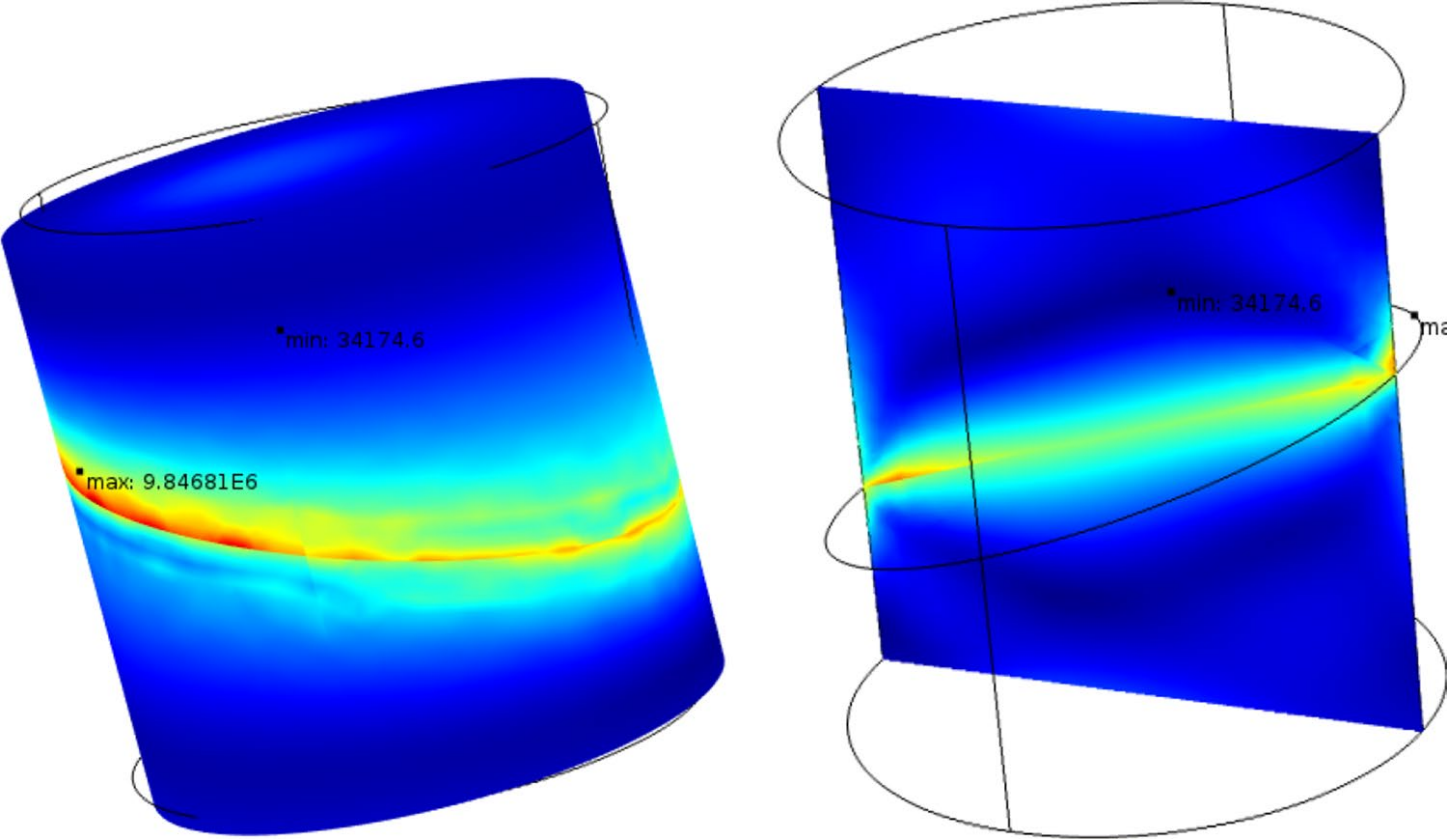
Von Mises stress distribution inside a digital object created to simulate the carbonate sample with the elastic property contrast. Left: 3D display of the stress distribution. Right: a 2D axial slice of the 3D model taken along the central axis. The boundary condition at the surface of the sample is uniform (hydrostatic) tension of 30 MPa.

The results shown in Fig. 4 indicate a sharp $\sigma_{VM}$ concentration at the boundary between the two domains with varying $E$. This is where the initial crack arguably developed as the sample was lifted from its original depth. Notice also the von Mises stress concentration at the circumference of the elastic discontinuity. Their location is where the minor cracks surrounding the main crack developed.

The stresses shown in Fig. 4 are in MPa, although any units could be used with the respective stress boundary conditions since the simulations were conducted under a linear elasticity assumption.

Of course, the same stresses but with the opposite sign would develop under hydrostatic compression. However, because rocks are weaker in tension than they are in compression (e.g.,^12^), still valid is the hypothesis that the “plane of weakness” in this sample developed during its extraction from depth, prior to laboratory experiments.

### Second sample: post-test and pre-test images

**Figure 5:**
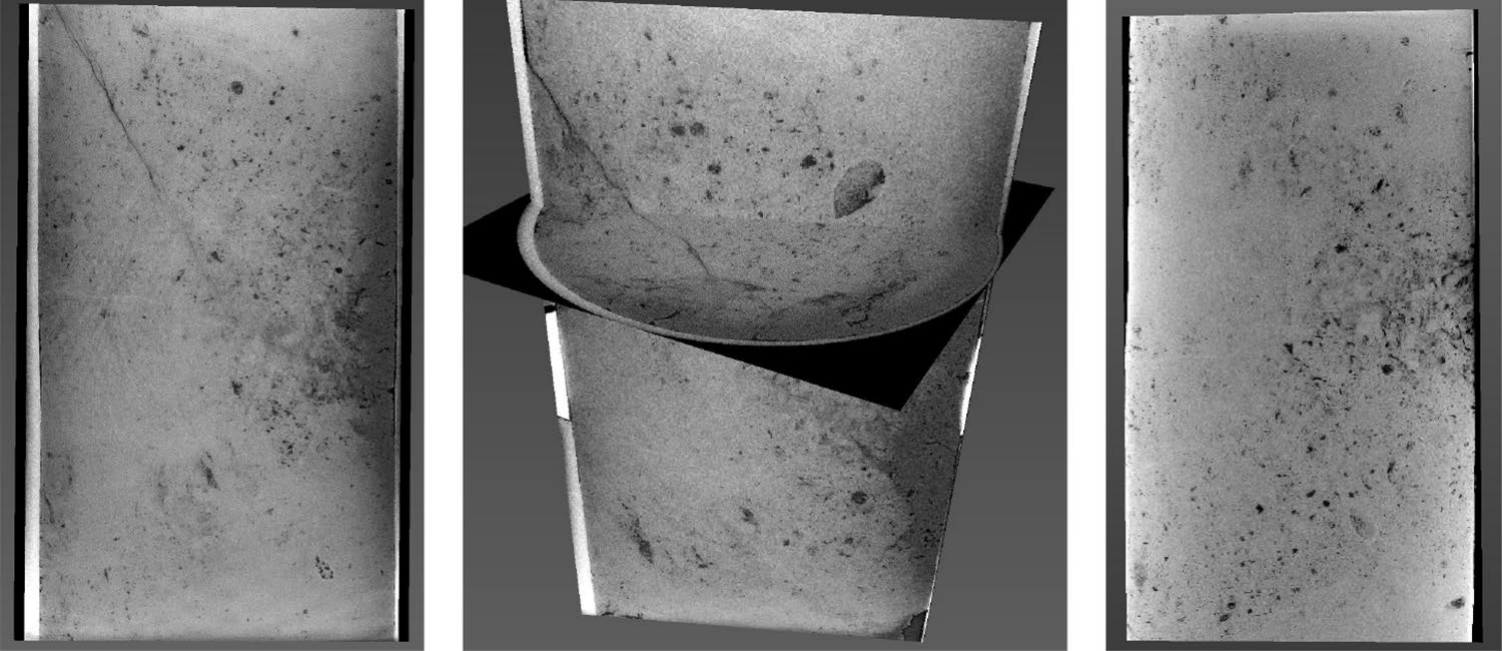
CT scan of C8 post-loading (first two images) and pre-loading (third image). The display is the same as in Fig. 3. The sample size is also the same as that of C23.

The post- and pre-test images for sample C8 are shown in Fig. 5. It also fractured, but unlike in the first sample, the crack is inclined at the textbook 45° to the horizontal.

There are also high-porosity darker strips visible in the pre-test image. However, unlike in C23, they are oriented at approximately 45° to the horizontal. Once again, an incipient crack might have developed along one of these discontinuities during sample recovery. Numerical tests, same as shown in Fig. 4, indicate that no matter how an elastic property discontinuity is oriented, sharp stress concentration appears along its trajectory under hydrostatic tension.

### Third sample: post-test and pre-test images

**Figure 6:**
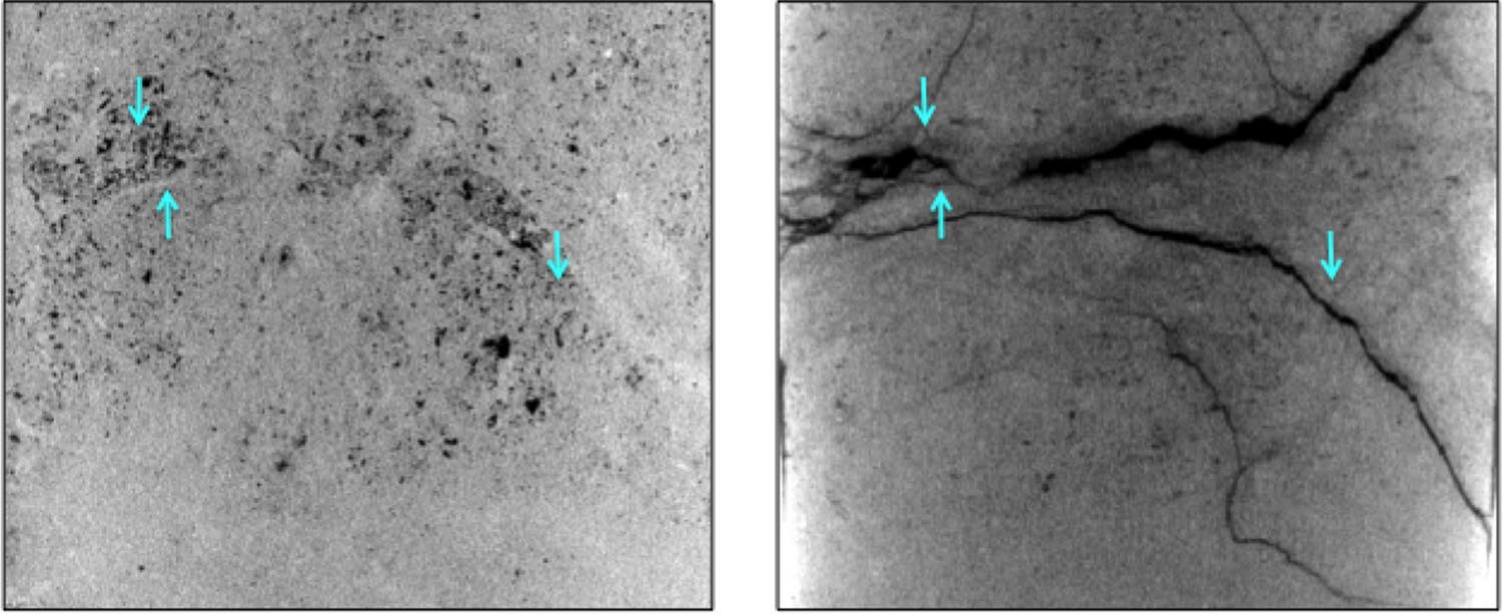
2D vertical slices along the central axis of 3D CT-scans of the third carbonate sample pre- (left) and post-testing (right). Arrows are used to point to the density and, as a result, elastic property contrasts in the pre-test image that developed into fractures clearly visible in the post-test image.

The hypothesis put forward here is further supported by the testing and respective pre- and post CT-scan images of the third carbonate sample. During the test, this sample’s behavior was qualitatively the same as that of the first two samples as shown in Fig. 2. A 3D CT-scan image taken after the test exhibited massive fracturing as shown in a 2D axial slice of the image in Fig. 6 (right). The locations of these fractures coincided with CT-intensity heterogeneity apparent in the pre-test image (Fig. 6, left). As in the first two samples, this intensity heterogeneity translated into density and elastic moduli heterogeneity, hence resulting in von Mises stress concentrations during tensile unloading of the core as it was evacuated from its original depth.

## Discussion and Conclusion

The effect of structural heterogeneity on crack initiation was discussed in^13^, where numerical modeling showed that the micro-heterogeneity played an important role in controlling both the micromechanical behavior and the macroscopic response when subjected to uniaxial compression loading. The crack-initiation stress was found to be controlled primarily by the micro-scale geometric heterogeneity. Earlier experimental results (e.g.^14^) also showed that microstructural heterogeneity plays a key role in creating local stress concentrations. Recently^15^, showed that grain boundary sliding can induce strain and rotation at the respective interfaces. In the new work reported here, a sample failure is observed as well, arguably due to tension-induced stress concentration along a structural heterogeneity.

It is evident from the CT-scan images in Figs. 3, 5, and 6 that the samples under examination contain relatively large vugs. A question arises whether the failure of a sample could have occurred due to the stress concentration around these features. A visual inspection of the aforementioned images indicates that this is not the case. Indeed, the fracturing occurred at the density contrast boundaries rather that at the vugs. To quantitatively assess the possibility of fracturing due to stress concentration at the vugs, we present here a simple model of an elastic body with isolated, as well as interacting spherical and elliptical vug-like inclusions.

**Figure 7:**
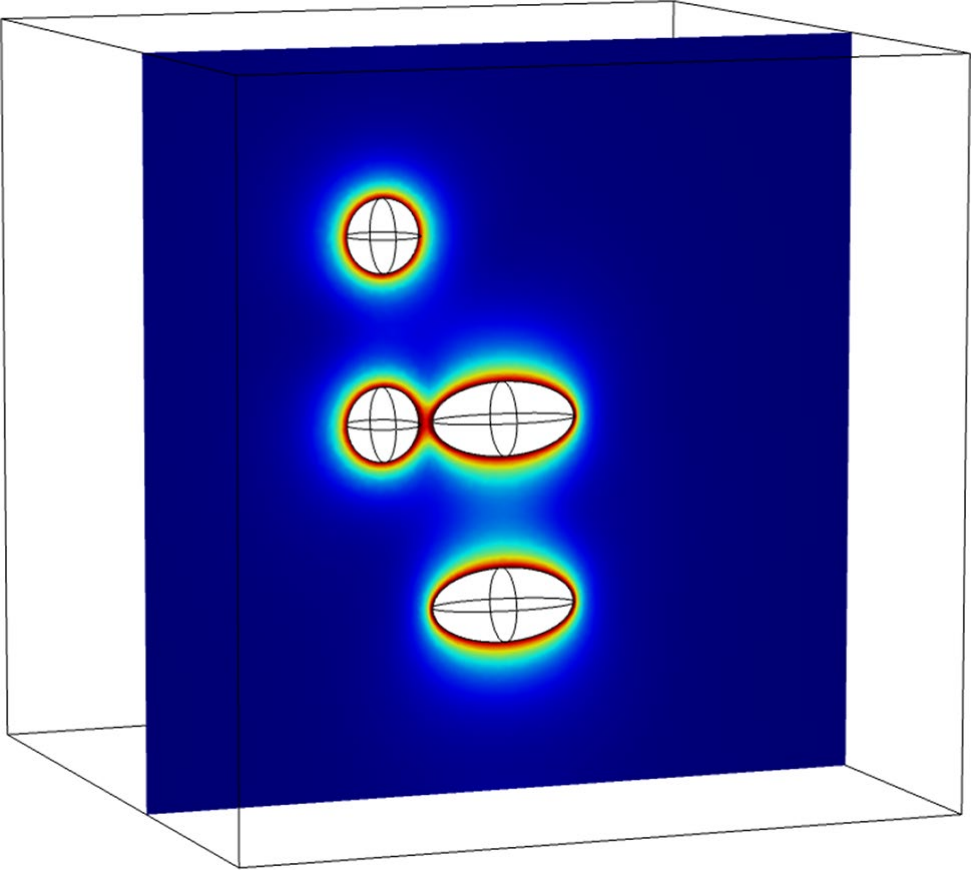
Von Mises stress distribution inside a digital object created to simulate elastic stresses around spherical and elliptical inclusions (vugs) under hydrostatic tension.

Figure 7 shows that stress concentrations under tension are localized around isolated inclusions as would be also evident from classic closed-form solutions^16^. Where the inclusions interact, once again, stress concentrations are localized around and between the inclusions and are unlikely to produce a plane of weakness cutting through the entire sample.

This work followed the classic scientific method: from the observation to a hypothesis to its validation to additional testing. Specifically, in trying to understand reasons for the occurrence of a subhorizontal (rather than inclined at 45°) crack in a rock sample subject to uniaxial loading, it was noticed that the sample exhibited a clearly visible density and structural discontinuity located exactly where the crack appeared. This discontinuity most likely translated into an elastic moduli contrast. The hypothesis is that an incipient fracture was generated along this discontinuity as the sample was lifted from the depth and unavoidably unloaded during this extraction. Later, during loading, the sample failed along this subtle plane of weakness. A numerical experiment confirmed that a strong von Mises stress concentration develops at an elastic property boundary during hydrostatic tension. This simulation confirms the hypothesis.

## Data Availability

The source data for Fig. 2 are available from the authors upon request.
